# Intermittent preventive treatment with sulphadoxine-pyrimethamine is effective in preventing maternal and placental malaria in Ibadan, south-western Nigeria

**DOI:** 10.1186/1475-2875-6-88

**Published:** 2007-07-06

**Authors:** Catherine O Falade, Bidemi O Yusuf, Francis F Fadero, Olugbenga A Mokuolu, Davidson H Hamer, Lateef A Salako

**Affiliations:** 1 Department of Clinical Pharmacology, University College Hospital, Ibadan, Tel. +234-803-326-4593, Nigeria; 2 Department of Pharmacology and Therapeutics, University of Ibadan, Ibadan, Nigeria; 3 Department of, Epidemiology Medical Statistics and Environmental Health, University of Ibadan, Ibadan, Nigeria; 4 Department of Pediatrics, Ladoke Akintola University of Technology, Oshogbo, Nigeria; 5 Department of Pediatrics, University of Ilorin, Ilorin Nigeria; 6 Center for International Health and Development, Boston University School of Public Health, Boston, MA, USA; 7 Section of Infectious Diseases, Department of Medicine, Boston University School of Medicine, Boston, MA, USA

## Abstract

**Background:**

Intermittent preventive treatment with sulphadoxine-pyrimethamine (IPT-SP) is currently the recommended regimen for prevention of malaria in pregnancy in endemic areas. This study sets out to evaluate the effectiveness of IPT-SP in the prevention of maternal and placental malaria in parturient mothers in Ibadan, Nigeria, where the risk of malaria is present all year round.

**Method:**

During a larger study evaluating the epidemiology of congenital malaria, the effect of malaria prophylaxis was examined in 983 parturient mothers. Five hundred and ninety eight mothers (60.8%) received IPT-SP, 214 (21.8%) received pyrimethamine (PYR) and 171 (17.4%) did not take any chemoprophylactic agent (NC).

**Results:**

The prevalence of maternal parasitaemia in the IPT-SP, PYR and NC groups was 10.4%, 15.9% and 17% respectively (p = 0.021). The prevalence of placental parasitaemia was 10.5% in the IPT-SP, 16.8% PYR and 17% NC groups, respectively (p = 0.015). The prevalence of maternal anaemia (haematocrit <30%) was 5.7% vs. 8.9% vs. 13.4% among the IPT-SP, PYR and NC groups respectively (p < 0.0001) while that of pre-term delivery (GA <37 weeks) was 10.5%, 19.2% and 25.3% among IPT-SP, PYR and NC groups respectively (p < 0.0001). Babies born to mothers in the IPT-SP, PYR and NC groups had mean birth weights of 3204 ± 487.16, 3075 ± 513.24 and 3074 ± 505.92 respectively (ρ < 0.0001). There was a trend towards a lower proportion of low birth weight babies in the IPT-SP group (p = 0.095).

**Conclusion:**

IPT-SP is effective in preventing maternal and placental malaria as well as improving pregnancy outcomes among parturient women in Ibadan, Nigeria. The implementation of the recently adopted IPT-SP strategy should be pursued with vigour as it holds great promise for reducing the burden of malaria in pregnancy in Nigeria.

## Background

The pregnant woman runs a higher risk of contacting malaria than her non-pregnant counterpart [1-4]. An estimated 25 million pregnancies are believed to occur annually in malarious areas of sub-Saharan Africa [5]. The transient depression of immunity to allow for development of the allograft (foetus) is one of the reasons adduced for the increased susceptibility of the pregnant woman to malaria. Although malaria in pregnancy is often asymptomatic, in the semi-immune woman, it nevertheless is the cause of unfavorable pregnancy outcomes both in the mother and in her baby [1,2,5-7]. The outcomes of the invasion of the placenta by parasites, inflammatory cells and cytokines include: abortion, premature labour, small-for-date babies and foetal/maternal death in some instances. These unfavorable pregnancy outcomes are associated with sequestration of malaria parasites in the placental intervillous spaces attached to chondroitin-sulphate-A [8,9]. Pro-inflammatory cells and cytokines also invade the placental bed. The net result is impairment of foetal blood and nutrient supply, which in turn predisposes to low birth weight (LBW). LBW, as occurs in small-for-date babies or babies born prematurely, is the greatest risk factor for neonatal mortality and a major contributor to infant mortality [9].

To prevent the adverse effects of malaria in pregnancy, antimalarial chemoprophylaxis is generally recommended. For a long time, prophylaxis with weekly pyrimethamine or chloroquine was widely adopted in many African countries [10]. Poor compliance and emergence of drug resistant strains of *Plasmodium falciparum *have, however, compromised the efficacy of these regimens [11,12]. Intermittent preventive treatment (IPT) with sulphadoxine-pyrimethamine (SP) after the first trimester has been found to be effective in reducing maternal anaemia, placental malaria and the incidence of LBW in studies in eastern and southern Africa [13-17]. This strategy involves giving a curative treatment dose of an effective antimalarial drug at predefined intervals during pregnancy. SP has been shown to be the most effective single dose antimalarial drug for prevention of malaria during pregnancy in areas where the strain of plasmodium remains sensitive to it [14,15]. Studies in Kenya, Mozambique and Malawi have shown that administration of at least two therapeutic doses of SP during the second and third trimesters of the pregnancy at an interval of 1 month apart is most effective in HIV-negative women [13,14,16,17]. Intermittent preventive therapy with SP (IPT-SP) is attractive because its single dose therapy lends itself for supervised administration in the antenatal clinic thus ensuring compliance.

Despite the well documented lack of efficacy of pyrimethamine for malaria chemoprophylaxis, many practitioners have continued to use this approach for pregnant women in Nigeria. This article reports the effectiveness of IPT-SP in the prevention of maternal and placental malaria in an urban hospital in Ibadan, Nigeria during a pilot study of the IPT-SP strategy.

## Patients and Methods

### Study site and ethical issues

The study was conducted at the St Mary's Catholic Hospital Eleta Ibadan between May 2003 and October 2004. St Mary's Hospital is a secondary health care facility run by the Catholic mission. It is located in the heart of the ancient city of Ibadan. However, St Mary's Hospital attracts patients of various socio-demographic classes from the entire city as a result of the high quality of service it has provided over decades at reasonable cost. Ibadan is located in the rain forest belt in south-western Nigeria. Malaria transmission is intense year round with a peak during the rainy season months of May to October and a nadir during the dry season months of November to April. In a recently conducted national efficacy study, day 14 efficacy of SP in Ibadan in acute uncomplicated malaria was 85% [18] among children aged 6 months to five years. The seroprevalence of HIV in the study area at the time of the study in adults (male and female) was 2% [19]. The University of Ibadan/University College Hospital Institutional Review Committee and the Boston University Institutional Review Board (collaborating institution) provided ethical approval for the study. Written informed consent was obtained from each study participant or her legal guardian (mother or husband) for those mothers less than 18 years of age.

### Study subjects, enrollment and laboratory procedures

Women who participated in a larger prospective observational study evaluating the epidemiology of congenital malaria in Ibadan, Nigeria were enrolled into the study [20]. Only patients who had resided in the catchment area for over two years, delivered live infants in the study hospital and provided signed informed consent were enrolled into the study. Enrolment was limited to 52 – 60 consecutive mother-baby pairs per month during the study period to cover both high and low transmission seasons. Booking clinics in St Mary's Hospital are held on Tuesdays and Thursdays while antenatal clinics are held on Mondays, Wednesdays and Fridays. At the beginning of the study, the standard operating procedure for malaria chemoprophylaxis was weekly pyrimethamine which was provided pre-packed for women to take at home every Sunday. Folic acid and iron supplements were also provided for pregnant women. The importance of malaria chemoprophylaxis and the use of haematinics (5 mg of folic acid daily and 200 mg ferrous sulphate thrice daily) is painstakingly explained to patients during health talks at the beginning of each booking and subsequent antenatal clinic visit.

Six months after commencement of the study, a pilot study on IPT-SP was introduced in some states in Nigeria. St Mary's Hospital was one of the hospitals selected for the IPT-SP pilot study in Oyo state. While other centers gave SP free to pregnant women, the study site gave IPT-SP at a cost of ₦50 ($0.35) per dose for sustainability. Three tablets of SP containing 500 mg sulphadoxine and 25 mg pyrimethamine per tablet (Melarich™, Medreich PLC England) were administered under supervision of midwives at the antenatal clinic and recorded in the patients' antenatal case notes. The use of IPT-SP was confirmed from the antenatal notes. Records were subsequently signed by the midwife responsible for its administration. Enquiries about untoward effects were made during the next clinic visit and were recorded. An investigator assisted questionnaire was administered to enrolled mothers at delivery to collect information on socio-demographic factors, malaria chemoprophylaxis, and occurrence of fever and malaria symptoms as well as anti-malaria drug use within two weeks of delivery. Details of other clinical and laboratory enrollment procedures are described in the report of the epidemiology of congenital malaria in Nigeria [20].

Babies born before 37 weeks of gestation were considered pre-term while those born from 37 weeks of gestation and above were considered term. Babies were weighed to the nearest gram using an electronic weighing scale. For the purpose of this study LBW was defined as neonatal birth weight less than 2,500 g while a haematocrit reading of <30% was considered as anaemia.

### Chemoprophylactic drug use

For the purposes of this study, all patients were categorized into three groups based on type of chemoprophylactic agent used. Patients in group 1 received IPT-SP, group 2 received pyrimethamine while those who took no chemoprophylactic agent were in group 3. Adequacy of IPT-SP use was defined as the use of at least two therapeutic doses of SP at least one month apart during the 2^nd ^and 3^rd ^trimester of pregnancy, where second trimester is defined as 14 to less than 28 weeks of gestation and 3^rd ^trimester as 28 weeks and above. Adequacy of pyrimethamine was defined as the use of 25 mg pyrimethamine weekly two weeks after quickening until term while the adequacy of chloroquine was defined as the use of 300 mg (two tablets) chloroquine base weekly for the same time duration as pyrimethamine. To be considered adequately used, proguanil needed to be taken at a dose of 200 mg daily for the same duration as pyrimethamine and chloroquine.

### Data analysis

Data collected were recorded into pre-coded case record forms. Thereafter, the data was double entered by two data entry clerks using EPI-INFO 6.04d (Centers for Disease Control and Prevention, Atlanta, GA, USA). Preliminary data analysis was done with EPI-INFO. Thereafter, the data were transferred to SPSS version 10 (SPSS Inc., Chicago, IL, USA) for analysis. Descriptive statistics such as means and standard deviations were used to summarize quantitative variables while categorical variables were summarized with proportions. Frequency tables and graphs were presented for relevant variables. The student t-test was used to compare two mean values while the one way analysis of variance (ANOVA) was used to compare mean values in more than two groups. The Chi-square test was used to investigate associations between two categorical variables and also to compare proportions. For significant associations, the odds ratio (OR) and 95% confidence intervals (CI) were computed. A p-value less than 0.05 was considered statistically significant.

## Results

Over an 18 month period (May 2003 to October 2004), 983 mother-baby pairs were enrolled. The mean maternal age was 29.6 ± 5.2 years. The youngest mother was 17 yrs of age while the oldest was 48 yrs. Only 11 (1.1%) of the mothers were less than 20 yrs of age. The socio-demographic characteristics of mothers in the IPT-SP, pyrimethamine (PYR) and no chemoprophylactic (NC) groups were similar (Table 1).

**Table 1:** Characteristics of parturient women who used IPT-SP, pyrimethamine or no chemoprophylactic agents

**Characteristics**	**IPT-SP Use (n = 589)**	**Pyrimethamine (n = 214)**	**No chemo-prophylaxis (n = 171)**	**p-value**
***Age (Years)***				
Mean ± SD	29.6 ± 5.7	29.4 ± 4.9	30.1 ± 5.3	0.408
Range	17 – 44	18 – 43	17 – 42	
***Maternal Level of Education ***n (%)				
None	13 (2.2)	3 (1.4)	4 (2.3)	
Primary/Qur'anic education	74 (12.4)	31 (14.5)	26 (15.2)	
Secondary school	359 (60.3)	122 (57.0)	85 (49.7)	0.307
Post 2^0 ^School	149 (25.0)	58 (27.1)	56 (32.7)	
**Total**	**595 (100)**	**214 (100)**	**171 (100)**	
***Fathers' Level of Education ***n (%)				
None	9 (1.5)	3 (1.4)	4 (2.5)	
Primary/Qur'anic education	53 (9.0)	21 (10.1)	19 (11.7)	
Secondary school	315 (53.5)	110 (53.1)	70 (43.2)	0.409
Post secondary school	212 (36)	73 (35.3)	69 (42.6)	
**Total**	**589 (100)**	**207 (100)**	**162 (100)**	
***Gravidity ***n (%)				
1	133 (22.2)	49 (22.9)	48 (28.2)	
2	127 (21.2)	50 (23.4)	35 (20.6)	0.516
>2	338 (56.5)	115 (53.7)	87 (51.2)	
***Baby's Sex ***n (%)				
Male	310 (51.8)	107 (50)	99 (57.9)	0.268

### Pattern of anti-vector use

The vast majority (956/983; 98.4%) of study participants admitted to using at least one form of anti-vector measure. Mosquito screens on windows were the most commonly used (77.9%) followed by insecticide sprays (69.1%), coils (25%), herbs (6.6%), untreated bed nets (6.6%) and mosquito repellants (0.5%). Insecticide treated bed nets (ITNs) were used by only 1.1% of pregnant women participating in the study.

### Pattern of antimalarial chemoprophylactic use

Most (840/983; 85.5%) mothers admitted to using some form of antimalarial chemoprophylaxis. The remaining 143 (14.6%) did not use any malaria chemoprophylactic drug during the index pregnancy. More than half of the mothers (598/983; 60.8%) received IPT-SP while 21.8% (214/983) received pyrimethamine monotherapy. Other chemoprophylactic agents used by mothers enrolled into the study include chloroquine (23/983; 2.3%), herbs (4/983; 0.4%) and proguanil (1/983; 0.1%). Five hundred and five of 598 (84.4%) patients who received IPT-SP received at least two therapeutic doses as stipulated in the guideline while 94.4% (202/214) of those who took pyrimethamine claimed to have taken adequate doses. All the patients who used chloroquine for chemoprophylaxis received grossly inadequate doses of chloroquine. The only patient who used proguanil took 100 mg daily for less than two weeks. All the patients who took chloroquine, herbal preparations and proguanil were considered as having taken no chemoprophylactic agent and were categorized into the NC group bringing the number in that group to 171 (17.4%).

### Malaria in the two weeks before delivery

A history of febrile illness in the two weeks preceding delivery was obtained in 9.5% (56/592), 23.5% (50/213) and 20.4% (34/167) of the mothers who received IPT-SP, PYR and NC respectively (p < 0.0001).

### Malaria parasitaemia

At delivery, the prevalence of maternal parasitaemia was significantly lower in the IPT-SP group when compared to the PYR and NC groups (Table 2). Both maternal and placental malaria parasitaemia were also significantly more prevalent among women who used pyrimethamine when compared with those who received IPT-SP (Table 3). There was no significant difference between the PYR and NC groups. The differences in the prevalence of patent parasitaemia in cord blood and in the peripheral blood of neonates (obtained by heel stick) born to mothers in the IPT-SP, PYR and NC groups were not statistically significant.

**Table 2:** Effects of IPT-SP, pyrimethamine or no chemoprophylactic agent on malaria parasitaemia and pregnancy outcomes in parturient women in southwestern Nigeria

**Outcome measure**	**IPT-SP**	**Pyrimethamine**	**No Chemoprophylaxis**	**P value**
	**N = 598**	**N = 214**	**N = 171**	
	**n (%)**	**n (%)**	**n (%)**	
***Maternal parasitemia***	62 (10.4%)	34 (15.9)	29 (17.0)	0.021
***Placental parasitemia***	63 (10.5%)	36 (16.8)	29 (17.0)	0.015
***Cord parasitemia***	16 (2.7%)	9 (4.2)	9 (5.3)	0.210
***Neonatal parasitemia ***	4 (0.7%)	3 (1.4)	1 (0.6)	0.553
***Maternal haematocrit ***(%)				
Mean ± SD	36.48 ± 4.55	36. 39 ± 4.7	35.17 ± 5.58	0.006
Range	18 – 45	20 – 45	17 – 45	
***Maternal anaemia***				
(Haematocrit <30%)	33/579 (5.7%)	19 (8.9)	26 (15.4)	<0.0001
***Pre-term delivery ***				
(Gestational age <37 weeks)	63 (10.5%)	41 (19.2)	43 (25.3)	<0.0001
***Neonatal birth weight(gm)***				
Mean ± SD	3204.3 ± 487.2	3075.7 ± 513.24	3074.7 ± 505.9	<0.0001
Range	1500 – 4700	1300 – 4500	1400 – 4500	
***Low birth weight ***(%)	31/595 (5.2)	17 (7.9)	16 (9.4)	0.095
***Neonatal haematocrit ***(%)				
Mean ± SD	58.2 ± 7.8	57.1 ± 7.66	56.8 ± 8.0	0.041
Range	35 – 79	34 – 75	31 – 75	
***Placental weight ***(gm)				
Mean 605.54 ± SD	605.5 ± 113.6	607.7 ± 131.9	588.9 ± 136.	0.253

**Table 3:** Risk of malaria parasitaemia and pregnancy outcomes among parturient women who received IPT-SP, pyrimethamine or no chemoprophylaxis in Ibadan, Nigeria

**Parameter**	**IPT-SP vs Pyrimethamine**			**IPT-SP vs No Chemoprophylaxis**		
	**OR**	**95% CI**	**p-value**	**OR**	**95% CI**	**p-value**

Maternal parasitaemia	0.612	0.39–0.96	0.036	0.57	0.35 9 091	0.019
Placental parasitaemia	0.582	0.37 – 0.91	0.02	0.577	0.36 – 0.93	0.022
Maternal anemia (Haematocrit <30%)	1.62	0.91 – 2.92	0.11	3.008	1.74 – 5.19	<0.0001
Pre-term delivery (GA <37 weeks)	2.03	1.31 – 3.31	0.002	2.88	1.84 – 4.35	<0.0001

### Maternal and neonatal haematocrit

The mean haematocrit of parturient women who used IPT-SP was higher than for the PYR and NC groups (p = 0.006) (Table 2). The mean haematocrit among mothers in the IPT-SP and the NC groups was significantly different (p= 0.002, 95% CI: -2.13 – -0.49) whereas there was no difference between the mean haematocrit of IPT-SP mothers and PYR mothers (p = 0.81). On the other hand, the prevalence of maternal anaemia was lower among IPT-SP users when compared to PYR and NC groups (ρ < 0.0001). Although there was no neonate with a haematocrit less than 30%, there was a statistically significant difference in the mean haematocrit of the three groups with the highest haematocrit in neonates whose mothers received IPT-SP (p = 0.041).

### Birth weight and pre-term delivery

The mean birth weight of babies born to mothers who received IPT-SP was significantly greater than those babies born to mothers in the PYR and NC groups (p < 0. 0001) (Table 2). When compared individually, babies born to mothers in IPT-SP group weighed significantly more than PYR and NC. The prevalence of LBW was found to be lowest among mothers who received IPT-SP when compared with mothers in the PYR and NC groups. However, these differences were not significant (p = 0. 095). Mothers who received IPT-SP were less likely to deliver pre-term babies than those who did not take IPT-SP (Tables 2 and 3). Preterm delivery occurred in 10.5%, 19.2% and 25.3% of mothers in IPT-SP, PYR and NC groups respectively (p < 0. 0001). The differences in the prevalence of preterm delivery among IPT-SP when compared specifically to pyrimethamine or NC groups were both statistically significant (Table 3).

### Safety evaluation

SP was well tolerated. There was no incidence of pruritus, fixed drug eruptions or allergic reactions attributable to any of the chemoprophylactic agents and IPT-SP used by any of the mothers. Five mothers reported episodes of dizziness within 30 minutes of taking SP. Dizziness in all cases terminated within a few hours without any specific treatment. There were no congenital malformations or deaths (maternal or neonatal) among the study participants.

## Discussion

Pregnancy-associated *P. falciparum *malaria is well recognized as an important cause of morbidity leading to maternal anaemia and LBW in the neonate [5-7,21]. The WHO currently recommends that each pregnant woman should receive at least two doses of IPT after quickening [5]. This recommendation is based on reports of beneficial effects of IPT-SP in preventing maternal and placental malaria and in improving pregnancy outcome in studies carried out in East Africa [13,14,16,17]. The findings of this study show that IPT-SP is effective in reducing the prevalence of maternal and placental malaria parasitaemia among parturient women in the urban city of Ibadan in southwestern Nigeria, an area hyperendemic for malaria. These findings are consistent with other reports in East and southern African countries [13,14,16,17] which all found IPT-SP protective against maternal and placental malaria. The results of this study are also in agreement with a report from Mali in West Africa which showed a protective effect of IPT-SP on the prevalence of maternal and placental malaria [22]. All of the aforementioned studies in other parts of Africa were randomized, controlled trials, whereas this study used an observational prospective design. Despite this major limitation, IPT-SP was highly effective in reducing maternal and newborn complications of malaria in pregnancy.

Challis et al [17] working in Mozambique reported a consistent reduction in the prevalence of malaria throughout pregnancy among mothers and also in the prevalence of placental malaria at parturition similar to the findings of the present study. Unlike the observation on maternal and placental malaria, the use or non-use of IPT-SP did not affect the prevalence of cord or neonatal malaria. Rogerson et al (2000) had earlier reported similar findings from Malawi. The incidence of cord and neonatal malaria was low with a cord parasite rate of 3.5% and congenital malaria rate of 0.8% in the study being reported here. In contrast to these findings, Challis et al [17] in their report noted a significant reduction in the incidence of congenital malaria among the neonates whose mothers used IPT-SP compared with placebo. Unlike the report of Challis et al there was no difference in the incidence of congenital malaria among babies born to mothers who received IPT-SP, PYR and NC. This may be due to the already low incidence of congenital malaria in the current study.

The differences in the prevalence of maternal and placental malaria among IPT-SP users and non-users occurred despite the fact that the vast majority of parturient women used one anti-vector measure or another. The pattern of anti-vector use was similar in both groups to a large extent. Although ITNs have been shown to be effective in the control of malaria in pregnant women [23,24], its use among pregnant women in the study area was very low (1.1%). The women who used no chemoprophylactic agent through out pregnancy consisted largely of patients who did not receive antenatal care at the study hospital and those who booked very late during the index pregnancy. Patients who used chloroquine, herbs, and proguanil were grouped into the same group as those who used no chemoprophylaxis because of poor compliance with these prophylactic agents. Resistance to chloroquine is also well established in the study area and many parts of sub-Saharan Africa [5,12,22]. The results of this study, which showed significant differences between IPT-SP and pyrimethamine users, provides further confirmation of the earlier reported compromised efficacy of pyrimethamine in preventing malaria in pregnancy [11]. The use of IPT-SP is clearly superior to pyrimethamine in protecting pregnant women from peripheral and placental parasitaemia, maternal anaemia and their neonates from LBW.

Judging from the marked reduction in the incidence of clinically diagnosed malaria in the two weeks preceding delivery in our study, IPT-SP use significantly reduced episodes of febrile illnesses suspected to be malaria during the last two weeks of pregnancy compared to mothers who used pyrimethamine or no chemoprophylaxis. Although it would have been useful to evaluate the timing of the last dose of IPT-SP prior to delivery, these data were not captured in the database of the study reported here.

### Effect of IPT-SP on LBW

LBW and prematurity are the greatest risk factors for neonatal mortality and a major contribution to infant mortality [7,9]. In this study, babies born to mothers who received IPT-SP on the average weighed more than babies born to those who used pyrimethamine and no chemoprophylactic agent respectively. There was also a trend towards a lower prevalence of LBW in the IPT-SP group. In addition, the prevalence of preterm delivery was also significantly lower among IPT-SP users when compared with those who took pyrimethamine.

### Effect of IPT-SP use on prevalence of maternal and neonatal anaemia

Anaemia is a well recognized consequence of malaria. Although maternal anaemia is multifactorial, malaria is known to contribute significantly to its occurrence in pregnancy. The prevalence of anaemia was least among parturient women who received IPT-SP during pregnancy when compared with the PYR and NC groups. Anaemia was earlier reported by workers in the same environment to have a negative impact on pregnancy outcome with still births occurring more often in anemic mothers [25]. Aimaku and Olayemi [25] also found that the birth weight and Apgar scores were significantly higher with increasing maternal haematocrit. The beneficial effect of IPT-SP on reduction of the prevalence of maternal anaemia is a welcomed finding which would lead to improved favorable pregnancy outcomes. The findings of this study are similar to another report from West Africa where IPT-SP was found to be protective against maternal anaemia, placental parasitaemia and LBW [22]. Unlike the Mali study however, the prevalence of maternal and placental malaria was much lower in our study. A prevalence of maternal and placental parasitaemia of 10.4% and 10.5% was recorded among IPT-SP users in our study compared to 21.8% and 24.5% in Mali. The Mali study was carried out in semi rural towns of Baudigana and Koro while the current study being reported was carried out in the urban city of Ibadan. Malaria transmission is more intense in rural than urban areas [26]. In addition, malaria transmission is perennial in Ibadan while it is seasonal in Bandiagana and Koro which might translate to lower acquired immunity among Malian women. Although the presence of malaria parasites in the placenta is a useful marker of the protective effect of malaria preventive measures in pregnancy, these findings are another reminder that measurement of impact indicators gives the best picture of efficacy and effectiveness. No baby in the study had a haematocrit below 31% and there was no significant difference between the mean haematocrit of babies born to mothers who received IPT-SP, pyrimethamine or the entire population of non IPT-SP users

### Safety and efficacy issues

Adverse events reported by study participants on IPT-SP and other chemoprophylactic agents were mild and did not necessitate any treatment. This is similar to the findings of previous workers [16,17,22]. Serious life-threatening allergic reactions to SP have also been reported to be relatively rare [27]. Concomitant administration of high dose folate supplementation (5 mg daily) as was done in St Mary's Hospital Eleta, the study site, was recently reported to compromise the efficacy of SP in a well designed clinical trial in Kenya [28]. It may be important to avoid high dose in favour of low dose (0.4 mg daily) folate supplementation in pregnant women receiving IPT-SP pending further elucidation of this drug-drug interaction.

## Conclusion

IPT-SP is highly effective in preventing maternal and placental malaria among parturient women in Ibadan, southwestern Nigeria as well as in improving pregnancy outcomes, including a lower prevalence of pre-term deliveries, bigger babies and lower prevalence of maternal anaemia. This study has once again confirmed the lack of efficacy of pyrimethamine for the prevention of maternal and placental malaria in pregnant Nigerian women [11]. The National Malaria Control Division of the Federal Ministry of Health recently adopted the policy of IPT-SP as part of the malaria control strategy in Nigeria [29]. The implementation of the recently adopted IPT-SP strategy if pursued with vigour holds great promise in reducing the burden of malaria in pregnancy in the country especially if combined with widespread use of insecticide-treated bed net among pregnant women.

## Authors' contributions

• Conception of the study and study design: CF, OM, DH

• Conduct of the study: CF, BY, FF, OM, DH

• Analysis of the data: BY

• Interpretation of the data: CF, BY, FF, DH, LS

• Drafting and critical review of the paper: CF, BY, FF, OM, DH, LS

• Read and approved final version of the paper: CF, BY, FF, OM, DH, LS
